# The effect of cell size and channel density on neuronal information encoding and energy efficiency

**DOI:** 10.1038/jcbfm.2013.103

**Published:** 2013-06-19

**Authors:** Biswa Sengupta, A Aldo Faisal, Simon B Laughlin, Jeremy E Niven

**Affiliations:** 1 Wellcome Trust Centre for Neuroimaging, University College London, London, UK; 2 Centre for Neuroscience, Indian Institute of Science, Bangalore, India; 3 Department of Bioengineering & Department of Computing, Imperial College, London, UK; 4 MRC Clinical Sciences Centre, Hammersmith Hospital Campus, London, UK; 5 Department of Zoology, University of Cambridge, Cambridge, UK; 6 School of Life Sciences and Centre for Computational Neuroscience and Robotics, University of Sussex, Falmer UK

**Keywords:** action potentials, energy metabolism, Na^+^ channels, K^+^ channels, mathematical modeling

## Abstract

Identifying the determinants of neuronal energy consumption and their relationship to information coding is critical to understanding neuronal function and evolution. Three of the main determinants are cell size, ion channel density, and stimulus statistics. Here we investigate their impact on neuronal energy consumption and information coding by comparing single-compartment spiking neuron models of different sizes with different densities of stochastic voltage-gated Na^+^ and K^+^ channels and different statistics of synaptic inputs. The largest compartments have the highest information rates but the lowest energy efficiency for a given voltage-gated ion channel density, and the highest signaling efficiency (bits spike^−1^) for a given firing rate. For a given cell size, our models revealed that the ion channel density that maximizes energy efficiency is lower than that maximizing information rate. Low rates of small synaptic inputs improve energy efficiency but the highest information rates occur with higher rates and larger inputs. These relationships produce a Law of Diminishing Returns that penalizes costly excess information coding capacity, promoting the reduction of cell size, channel density, and input stimuli to the minimum possible, suggesting that the trade-off between energy and information has influenced all aspects of neuronal anatomy and physiology.

## Introduction

Since the work of Golgi and Cajal,^1, 2^ it has been clear that the somata, dendrites, and axons of neurons vary enormously in size. Size differences among neuron classes within the nervous system can be substantial,^3, 4^ as can differences in homologous neurons among species of different sizes^5^ and the same neurons during development. While ion channel noise appears to set a fundamental lower limit to soma and axon diameters,^6^ the reasons why there is such variation above this limit remain unclear. Factors, such as conduction velocity or the numbers of synapses received and made, are related to the size of some neural components but not all. For example, differences in axon diameter are often attributed to the need for different conduction velocities; larger diameters conducting at higher velocities.^7, 8^ However, a recent survey of 16 tracts from five species suggests that conduction velocity cannot explain variation in axon diameter, which may be better explained by the information rate, with axons constrained to deliver information at the lowest acceptable rate because of the costs of excess capacity.^4^

Changes in the size of neurons affect their electrical properties and, consequently, their information coding. The electrical properties of neurons are determined by a combination of factors such as passive membrane properties, active conductances, the shapes of dendrites and axons, and the locations of ligand- and voltage-gated ion channels. Changes in the size of electrical compartments will alter the area of bi-lipid membrane and, consequently, the total membrane capacitance. Changes in size will also affect the densities of voltage-gated ion channels altering the amount of channel noise, the spike threshold, and the maximum attainable spike rate.^9, 10, 11^ These two factors, capacitance and channel density, will interact to influence the way in which the membrane filters synaptic or sensory inputs and, therefore, the information-processing capabilities of neurons. For instance, assuming the specific capacitance and the input resistance as constant, a larger passive cell would have a lower bandwidth in comparison to its smaller counterpart. The importance of the relationship between compartment size and information coding is supported by experimental studies of the mammalian optic nerve that have suggested the majority of information is transmitted at low rates in narrow fibers.^12, 13^

Changing channel density and total membrane capacitance will also affect the energy expended by neurons on restoring ion gradients across their membrane.^14^ Work on fly retina has shown that metabolic consumption is dependent upon photoreceptor size; smaller photoreceptors consume an order-of-magnitude less energy than larger photoreceptors.^15^ Moreover, larger photoreceptors with higher information rates also have lower energy efficiencies, producing a Law of Diminishing Returns that penalizes excess information capacity. Because fly photoreceptors, like their vertebrate counterparts, are graded potential neurons, the extent to which the relationships between neuron size, information rates, and energy consumption are similar in single spiking neurons is unclear. Moreover, experimental studies cannot easily explore the limitations that relationships between information coding and energy consumption impose on neuronal size because the functional neurons do not exceed biophysical and biochemical limits.

Here we study the role of compartment size and channel density using single compartment models of different sizes that incorporate stochastic voltage-gated Na^+^ and K^+^ channels from squid giant axon and are stimulated by excitatory synaptic inputs. By assessing the performance (bits s^−1^), energy consumption (ATP s^−1^), and energy efficiency (bits ATP^−1^) of compartmental models, we show that the largest compartments have the highest information rates but the lowest energy efficiency for a given voltage-gated ion channel density. The largest compartments also have the highest signaling efficiency (bits spike^−1^) for a given firing rate. Each compartment shows a maximum efficiency with channel densities between a half and a quarter that of the squid giant axon. However, maximum information rates were achieved at channel densities between a half and one times that of the squid giant axon irrespective of the size of the compartment. Thus, the largest compartments have the highest performance but incur a severe metabolic cost because of reduced energy efficiency, penalizing excess information processing capacity. Our models suggest that it is more energy efficient for information to be encoded at lower rates in many small neurons rather than a few large, high-information-rate neurons.

## Materials and Methods

### Single Compartment Model with Conductance Noise

We simulated single-compartment models containing stochastic voltage-gated ion channels, the properties of which were based on the original Hodgkin–Huxley model of a squid axon.^16, 17^ The model contained transient voltage-gated Na^+^ channels and delayed rectifier voltage-gated K^+^ channels along with a non-probabilistic voltage independent Leak conductance. The dynamics of the membrane potential was governed by the following current balance equation:


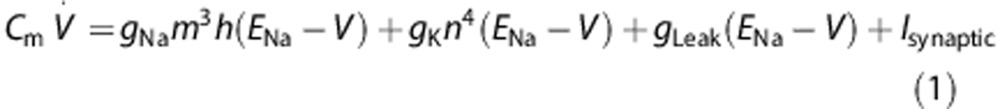


where *C*_m_ is the membrane capacitance, *g*_Na_, *g*_K_ and *g*_Leak_ are the conductances of the Na^+^, K^+^, and Leak channels, respectively. *E*_*j*_'s are the reversal potentials of these conductances, where 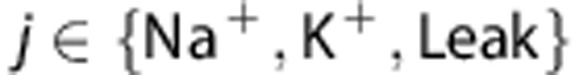. The variables *m*, *h*, and *n* follow first-order kinetics of the form 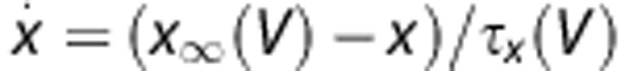, where 
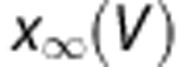
 is the steady-state activation or inactivation and 
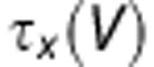
 is the voltage-dependent time constant. 
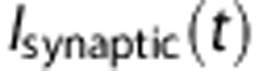
 is a time-dependent synaptic current. In our simulations, 
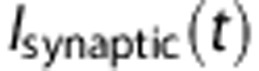
 was excitatory and noise-free, as we did not model input noise.


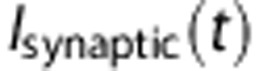
 was modeled as an excitatory conductance stimulus.^18^ We describe the dynamics of 
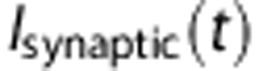
 by taking the example of a single excitatory conductance source, 
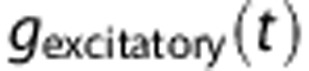



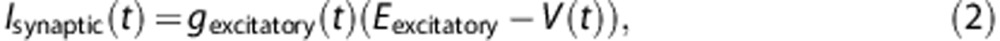


where 
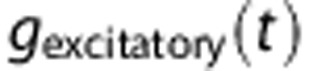
 follows the following differential equations,


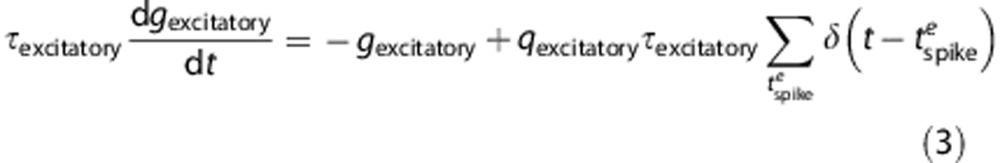



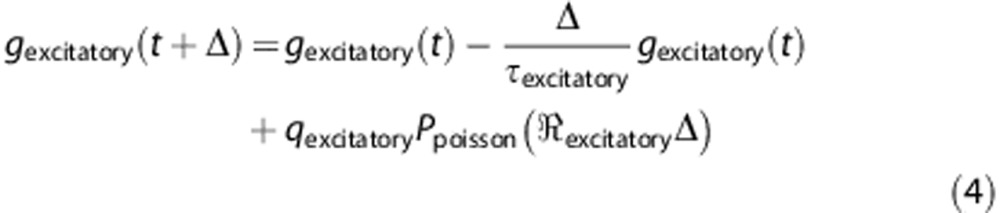


Equation (4) is a discretized version of equation (3), where 
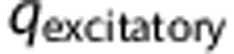
 defines the unitary quantal conductance increase due to a single presynaptic spike. The increase in conductance decreases exponentially with a time constant, 
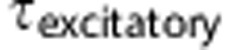
. In equation (4), 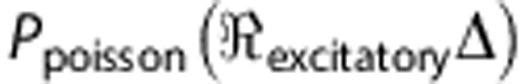 defines the number of spikes that arrive within a time-step, Δ, with a Poisson distribution having a mean rate of 
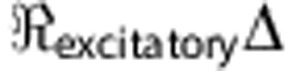
.

The stimulus was presented for 1 second and each set of simulations consisted of 60 such trials. All Gaussian random numbers were generated using the Marsaglia's Ziggurat algorithm; uniform random numbers were generated using Mersenne Twister algorithm. Deterministic equations were integrated using the Euler algorithm while stochastic differential equations were integrated using the Euler–Maruyama method, both with a step size of 10 microseconds. Parameter values are given in Supplementary Table S1. Markov state transitions for the voltage-gated ion channels are modeled after the channel noise formulation in.^9, 16^

### Scaling of Channel Densities

To study the effect of channel densities upon metabolic efficiency of information processing, we scaled the channel densities of the voltage-gated Na^+^ and K^+^ channels while keeping the ratio constant. The original Hodgkin–Huxley model of the squid giant axon has 60 Na^+^ channels and 18 K^+^ channels for every *μ*m^2^ of membrane. Therefore, a 100 *μ*m^2^ membrane contains 6,000 Na^+^ and 1,800 K^+^ channels. If the absolute density is doubled, the 100 *μ*m^2^ membrane now contains 12,000 Na^+^ and 3,600 K^+^ channels.

### Information Rates for Spiking Neuron Models

We used the ‘direct method' to measure the entropy of the responses,^19^ which compares different spike trains without reference to the stimulus parameters. The total entropy sets the information capacity for the spike train. The noise entropy measures the variability of the spike train across trials. These quantities were dependent upon the temporal resolution with which the spikes were sampled, 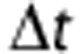 (1 millisecond), and the size of the time window, *T*. We presented either a different conductance trace in each subsequent trial (unfrozen noise) to calculate the total entropy, or the same conductance trace in each subsequent trial (frozen noise) to calculate the noise entropy. We divided the spike train to form *K*-letter words (*K*=2, 4, 6, 8, 12, 16, 24, 32, 48, or 64), where 
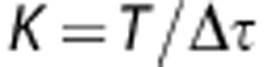
. We used the responses from the unfrozen noise presentations (60 trials each of 1 second) to estimate the probability of occurrence of a particular word, *P(W)*. The total entropy was estimated as


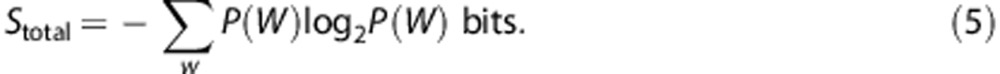


We estimated the probability distribution of each word at specified time durations, *t*, to obtain the conditional probability *P(W|t)*. Entropy estimates were then calculated from these distributions and the average of the distributions at all times computed to give the noise entropy (60 trials each of 1 second) as


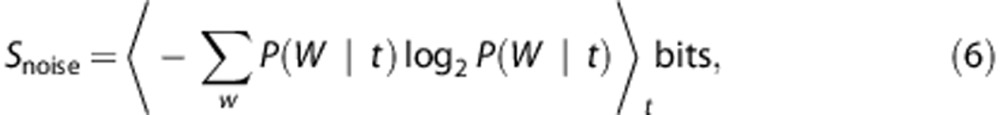


where 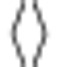 indicates average over time. The information was then computed as


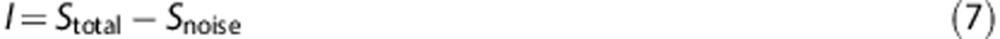


The total entropy and the conditional noise entropy diverge in the limit 
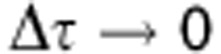
, their difference converges to the true finite information rate in this limit.^19^ Therefore, we used bias correction methods to reduce the effect of sampling errors.^20^ Using Δ*t*=1 millisecond, we varied the spike trains to form words of different lengths. Using these entropy estimates, we extrapolated to infinite word length from the four most linear values of the entropy versus the inverse word length.

### Calculating Energy Consumption

The energy consumption of each compartmental model is determined by the amount of trial-averaged ATP molecules expended in a single second (averaged over 60 trials of 1 second each), either without a stimulus or during the encoding of the band-limited conductance waveform. The Na^+^/K^+^ pump hydrolyzes one ATP molecule for three Na^+^ ions extruded and two K^+^ ions imported.^21, 22^ We determined the total K^+^ current by separating the Leak current into a K^+^ permeable Leak current and adding it to the delayed rectifier K^+^ current. We computed the number of K^+^ ions by integrating the area under the total K^+^ current curve for the duration of stimulus presentation. We calculated the number of ATP molecules used by multiplying the total K^+^ charge by *N*_A_/(2*F*), where *N*_A_ is Avogadro's constant and *F* is Faraday's constant.

## Results

### Model Description

**Figure 1:**
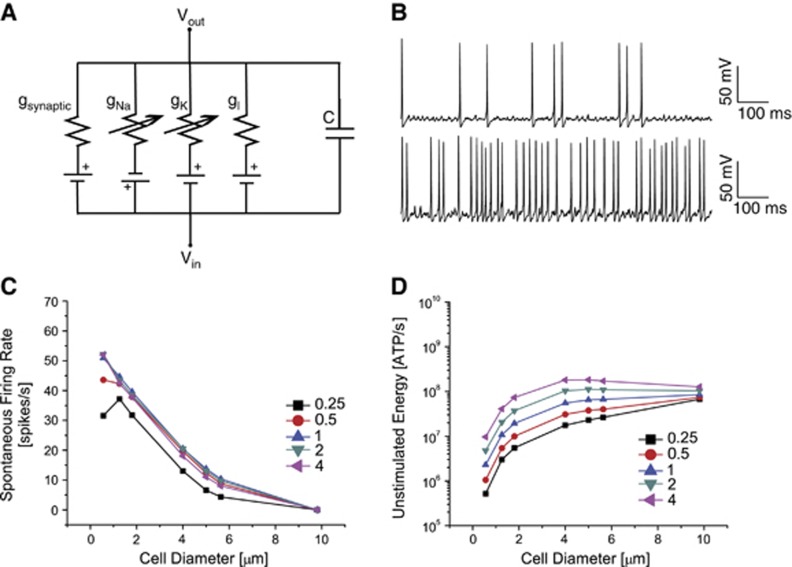
The single-compartment model. (**A**) Single-compartment model with two voltage-gated conductances, *g*_Na_ and *g*_K_, and a Leak conductance, *g*_l_. The model has a capacitance, *C*, determined by the size of the compartment and receives excitatory synaptic inputs, *g*_synaptic_. (**B**) Top: An example of spontaneous action potentials triggered by channel noise in a 100 *μ*m^2^ compartment with the same density of voltage-gated ion channels as the original squid axon model. Bottom: An example of spontaneous action potentials triggered by channel noise in a 10 *μ*m^2^ compartment with half the density of voltage-gated ion channels as the original squid axon model. (**C**) The rate of spontaneous action potentials in cells varying from 0.6 to 9.8 *μ*m in diameter (equivalent to 1 to 300 *μ*m^2^ compartments) for channel densities ranging from a quarter of to four times the density of voltage-gated Na^+^ and K^+^ channels found in the squid giant axon. There is no excitatory input stimulus. (**D**) The corresponding metabolic energy consumption for the compartment sizes and channel densities shown in **C**. Colors and symbols indicate the density of the voltage-gated ion channels.

We simulated single-compartment isopotential models containing stochastic voltage-gated Na^+^ and K^+^ channels and an additional voltage independent Leak conductance (Figures 1A and B). We changed the area of the compartment systematically from 1 to 300 *μ*m^2^, equivalent to a change in the diameter of a spherical compartment of 0.6 to 9.8 *μ*m. We also altered the density of the voltage-gated Na^+^ and K^+^ channels from the original numbers found in the Hodgkin–Huxley model of squid giant axon, which contains 60 Na^+^ and 18 K^+^ channels in each *μ*m^2^ of membrane.^16, 17^ For each compartment, both channels' densities were changed simultaneously, altering the absolute numbers of ion channels but not their relative proportions (see Materials and Methods).

### Spontaneous Activity and Energy Consumption

In the initial simulations, we assessed the ionic currents and the resulting membrane potentials generated by channel noise in these compartments in the absence of synaptic inputs (Figure 1B). In the smallest compartments, the spontaneous opening of small numbers of voltage-gated Na^+^ channels due to thermal agitation depolarizes the compartment sufficiently to evoke action potentials. The rate of spontaneous action potentials declines as the compartments become larger until the firing rate approaches 0 for the 300 *μ*m^2^ compartment, regardless of the channel density (Figure 1C); with increasing numbers of voltage-gated channels in larger cells, spontaneous single-channel openings make too small a contribution to depolarize the compartment, increasing the current threshold for action potential initiation. In compartments of 100 *μ*m^2^ or less, where the current threshold for firing is lower, increasing the channel density increases the spontaneous firing rate (Figure 1C). Very low channel densities, a quarter of the original density, reduce the spontaneous spike rate irrespective of the compartment size because there are approximately four times fewer voltage-gated Na^+^ channels capable of opening to trigger an action potential.

Action potentials consume energy because Na^+^ and K^+^ ions that flow across the membrane during the action potential must be restored by the Na^+^/K^+^ pump, requiring ATP.^23^ Yet, despite the smallest compartments having the highest spontaneous spike rates,^6, 24^ larger compartments consume more energy in the absence of synaptic inputs, irrespective of the channel density (Figure 1D). This can be explained by the total ion flux during an action potential, which is lower in smaller compartments with fewer ion channels than in larger compartments with greater numbers of ion channels. Likewise, lower channel densities reduce costs, even when they do not affect the spontaneous spike rate, because each action potential opens fewer channels consuming less energy (Figure 1D).

In the largest compartments, with low or zero spike rates in the absence of synaptic inputs, the majority of the energy is consumed to maintain the resting potential rather than by spontaneous action potentials. In large compartments, the large ion flux at rest can equal or even exceed the costs of spontaneous action potentials in smaller compartments (Figure 1D). For example, in the absence of synaptic inputs, 9 Na^+^ channels are open in the 300 *μ*m^2^ compartment versus a single channel in the 1 *μ*m^2^ compartment, and 49-times-more K^+^ channels are open in the 300 *μ*m^2^ compartment than in the 1 *μ*m^2^ compartment. Increasing channel density increases the cost of resting potential maintenance. For example, a 300 *μ*m^2^ compartment with a quarter-fold the original density of ion channels opens, on average, 29 K^+^ channels while the same compartment with four-fold the original density opens, on average, 133 K^+^ channels. The four-fold increase in channels also causes an, on average, ∼13-fold increase in the numbers of open Na^+^ channels. At the highest channel densities, the resting energy consumption in the large compartments equals the energy consumption of action potentials in smaller, spontaneously active compartments. In the largest compartments, the resting energy consumption approaches that of a non-probabilistic model because of the presence of large numbers of voltage-gated ion channels.

The metabolic cost has two components, ion flux through the voltage-gated channels and the Leak conductance. The importance of the Leak conductance cannot be understated; in a 300 *μ*m^2^ compartment with four-fold the original density, the total energy consumption increases by 19% when the energy consumed by the Leak conductance is added to that consumed by the voltage-gated K^+^ channels (Supplementary Table S2). At lower channel densities, the contribution of the Leak is even more significant; in a 300 *μ*m^2^ compartment with the original density of channels, the Leak increases the resting energy consumption by 122%, while in a 1 *μ*m^2^ compartment the Leak inflates energy consumption by 42%. Indeed, at low channel densities the cost of ion flux through voltage-gated channels diminishes and the cost of the Leak conductance is the primary determinant of energy consumption.

### Firing Rates and Information Coding

**Figure 2:**
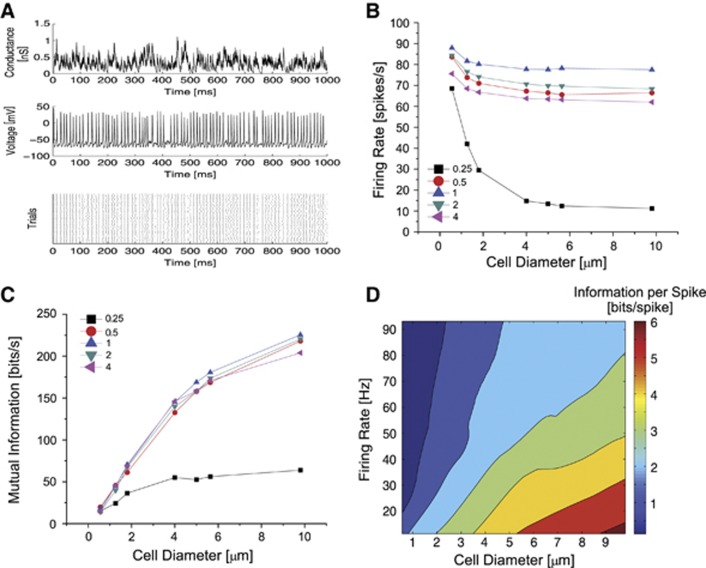
The influence of compartment size and channel density upon firing rate and information coding. (**A**) Top: An example of the excitatory conductance stimulus. Middle: An example of a spike train evoked by the conductance stimulus. Bottom: Spike rasters across 60 trials in response to presentations of identical conductance waveforms. (**B**) The spike rates in model cells receiving excitatory synaptic inputs for voltage-gated Na^+^ and K^+^ channel densities from a quarter of to four times the density of those found in the squid giant axon. Cells varied in diameter from 0.6 to 9.8 *μ*m (equivalent to 1 to 300 *μ*m^2^ compartments). Colors and symbols indicate the density of the voltage-gated ion channels. (**C**) The information rates of each of the model cells shown in **B**. (**D**) A contour plot showing how the coding efficiency in bits/spike depends upon cell diameter and firing rate for all the compartments modeled (including those in Supplementary Figures S1 and S3). The data points have been fitted using locally weighted linear regression.

The compartments were stimulated with excitatory synaptic inputs at 500 Hz, their intervals drawn from a Poisson distribution (Figure 2A). Each synaptic input caused a conductance change of 0.2 mS/cm^2^ so that the synaptic input scales in proportion to the membrane area. This scaling of synaptic input is equivalent to increasing the number of identical excitatory synapses that the compartment receives. The inputs are noise-free so the voltage-gated channels remain the only noise source in our simulations. The synaptic inputs were sufficient to evoke spikes in all the compartments (Figure 2A), though their rates varied from 88 spikes/second in the 1 *μ*m^2^ compartment at the original channel density to just 11 spikes/second in the 300 *μ*m^2^ compartment at a quarter of the original channel density (Figure 2B).

When driven by excitatory synaptic inputs, the smallest compartments have the highest firing rate (Figure 2B) because of the contribution of spontaneous action potentials (Figure 2A). As the compartment size increases, the rate drops initially but plateaus at cell diameters of 4 *μ*m because the membrane filtering properties reduce the impact of single-channel conductances. Altering the channel density from the original Hodgkin–Huxley model reduces the firing rate, irrespective of the compartment size (Figure 2B). At low channel densities, this is caused by insufficient numbers of voltage-gated Na^+^ channels being available to support spikes. However, at high channel densities, this is due to the presence of high numbers of voltage-gated K^+^ channels; models with a two- or four-fold higher densities of Na^+^ channels with the density of K^+^ channels fixed at 18/*μ*m^2^ show no reduction in firing rate (Supplementary Figure S1).

We measured the spike train entropy generated by frozen and unfrozen synaptic inputs (see Methods). Using frozen inputs, where the same 1-second-long input is repeated 60 times, we evaluated spike train reproducibility, enabling us to obtain the noise entropy. Using unfrozen inputs, we quantified the total entropy that is representative of the repertoire of spiking patterns that can be produced by the compartment. Spike trains generated by the smallest compartments have the highest total entropy (Supplementary Figure S2A). Increasing compartment size reduces the total entropy up to 50 *μ*m^2^, beyond this the total entropy plateaus because large compartments display limited spike time variability. For a given compartment size, increasing or decreasing the ion channel density from that of the original model reduces the total entropy of neural coding. These trends in total entropy closely resemble those for the firing rate across the compartment sizes and channel densities.

Smaller compartments have higher noise entropy than the larger compartments (Supplementary Figure S2B). Increasing the compartment size decreases the noise entropy, irrespective of channel density. The decrease is most marked at low channel densities, and is due to fluctuations in small number of channels with comparatively large discrete conductances, which increases the effect of channel noise. For all but the lowest channel densities, the decrease in noise entropy is larger than the decrease in signal entropy. For example, for compartments with the original ion channel density, increasing compartment size from 1 to 300 *μ*m^2^ decreased the total entropy by 8% but the noise entropy by 66%.

The difference between the total and the noise entropies is the mutual information (MI), a direct measure of the amount of information in the spike train free of assumptions about how the information is represented and what it means.^19^ The MI increases with compartment size, irrespective of the channel density (Figure 2C). Although our simulations show that the highest information rates are achieved at the original channel density, the differences in information rate are small for all but the lowest channel density (c.f. Schneidman *et al*^25^). Because compartments of different sizes differ in their spike rates, we quantified the information per spike for all our models, including those in which the relative proportions of voltage-gated Na^+^ and K^+^ channels differed (Supplementary Figure S3). Larger compartments encode more information per spike when the comparisons are made at similar firing rates (Figure 2D). For example, at 50 spikes/second, an 8 *μ*m (3.5 bits/spike) diameter compartment codes 137% more information per spike than a 2 *μ*m (1.5 bits/spike) diameter compartment and 22% more information per spike than a 5 *μ*m (2.8 bits/spike) diameter compartment.

### Energy Consumption and Energy Efficiency

**Figure 3:**
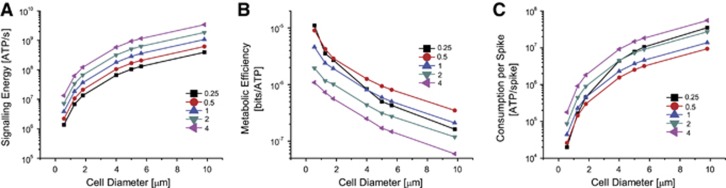
The influence of compartment size and channel density upon energy consumption and information coding efficiency. (**A**) The metabolic energy consumption of each of the compartments for voltage-gated Na^+^ and K^+^ channel densities from a quarter of to four times the density of those found in the squid giant axon. Cells varied in diameter from 0.6 to 9.8 *μ*m (equivalent to 1 to 300 *μ*m^2^ compartments). (**B**) The energy efficiency of each of the model cells shown in **A**. (**C**) The energy consumption per spike of each of the model cells shown in **A**. Colors and symbols indicate the density of the voltage-gated ion channels.

Larger compartments incur higher metabolic costs while signaling, regardless of the density of Na^+^ and K^+^ channels, the metabolic costs scaling close to the membrane area (Figure 3A). Compartments with more channels also consume more energy than those with fewer channels; at four-fold the original channel density energy consumption is an order of magnitude greater than at a quarter-fold. Greater channel numbers allow more ions to flow across the membrane during an action potential. Thus, action potentials in large compartments or those with higher channel densities consume more energy because the Na^+^/K^+^ pumps must do more work to restore the ion gradients.

We calculated the energy efficiency of coding by dividing the energy consumption during signaling with the corresponding information rates (Figure 3B). The energy efficiency drops as the compartment size increases for all channel densities. The energy efficiency also drops as channel density increases, except at the lowest densities; at these densities, information rates are substantially lower than at other densities, except at the smallest sizes. Therefore, for each compartment size, there is a density of Na^+^ and K^+^ channels that maximizes the energy efficiency. In our models, this is half the density of the original model, except at the smallest compartment size (Supplementary Figure S4). Comparing the 1 and 300 *μ*m^2^ compartments, we see that energy efficiency falls because consumption increases roughly in proportion to membrane area from 2.2 × 10^6^ to 6.2 × 10^8^ ATP molecules/second (Figure 3A) whereas information rate increases by a more modest amount from 20 to 218 bits/second (Figure 2C). Note that for all but the smallest compartment, the minimum energy consumption per spike occurs at half the original channel density (Figure 3C).

**Figure 4:**
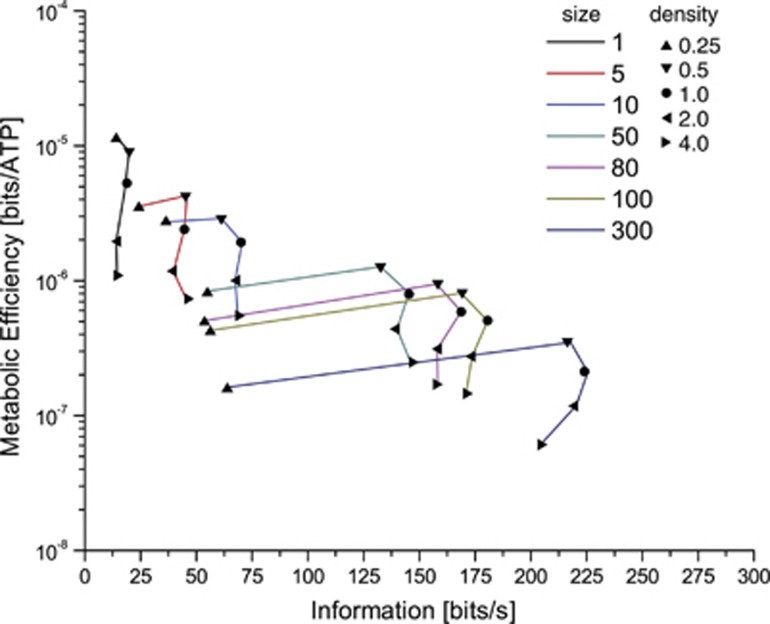
Channel density affects the trade-off between information processing and metabolic efficiency. As compartments increase in size from 1 to 300 *μ*m^2^, the information rates they achieve increase but metabolic efficiency drops for a given channel density. Voltage-gated Na^+^ and K^+^ channel densities ranged from a quarter of to four times the density of those found in the squid giant axon. Colors indicate compartment size while symbols indicate the density of the voltage-gated ion channels.

To investigate the trade-off between performance and efficiency, we plotted the information rates of each compartment at each channel density against the energy efficiency (Figure 4). Smaller compartments are more energy efficient than the larger compartments with the same channel density, but have lower information rates. For each compartment, except the smallest, the maximum efficiency occurred at half the original channel density. Higher information rates are attainable at higher channel densities but only by sacrificing energy efficiency (Figure 4). Consequently, to code between 80 and 140 bits/second, the most efficient single compartment would be the 50 *μ*m^2^ compartment with half the original channel density. However, to code 150 bits/second, the most energy efficient solution would be the 50 *μ*m^2^ compartment with the original channel density. Increasing the channel density beyond the original numbers only deteriorates the energy efficiency without further improving the information rate.

Thus, there is a Law of Diminishing Returns whereby increasing compartment size increases the information rate but reduces the energy efficiency (Figure 4). Changes in channel density shift the trade-off between performance and efficiency within a given compartment size, but cannot make a compartment more efficient than the smallest compartment that can operate at a particular information rate. Thus, the compartment size should be reduced to the minimum possible to encode a particular information rate.

### Stimulus Statistics

**Figure 5:**
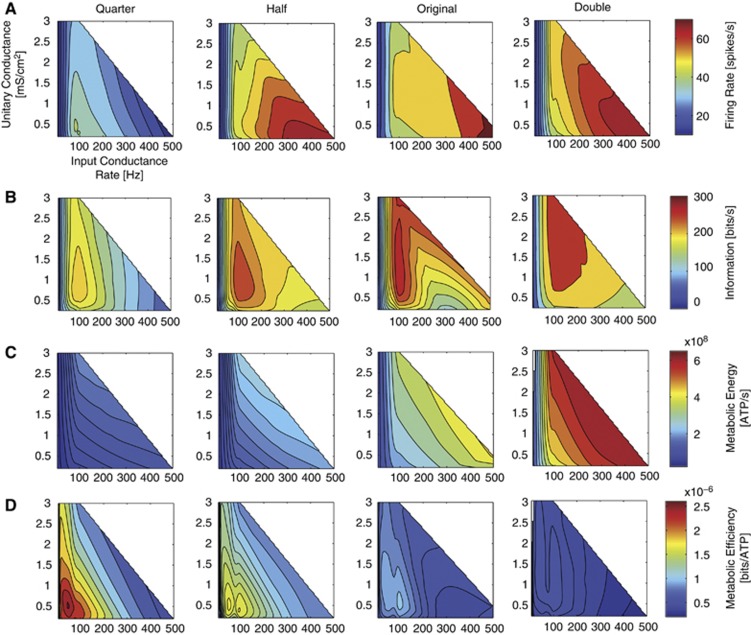
The effect of the input stimulus statistics on the information processing and energy efficiency of a 100 *μ*m^2^ compartment. Each panel shows the rate of conductance input versus the amplitude of the unitary conductance input. The color contours in each row indicate the (**A**) firing rates, (**B**) information rates, (**C**) metabolic energy, and (**D**) metabolic efficiency. A scale is given at the right hand side of each row. Each column shows a different density of voltage-gated Na^+^ and K^+^ channel densities from a quarter of to two times the density of those found in the squid giant axon. The white areas in each plot indicate the region in which input stimuli were not simulated.

To determine how the relationships between size, channel density, information coded, and energy consumed are affected by the input stimulus statistics, we changed the input rates and unitary amplitudes of the excitatory synaptic inputs systematically (Figure 5) (see Methods). The densities of voltage-gated Na^+^ and K^+^ channels changed as in our previous simulations (see Methods) but the compartment's diameter was kept at 5.6 *μ*m (100 *μ*m^2^) throughout these simulations.

At all but the lowest channel density, as the rate of synaptic inputs increases, the firing rate also increases. The original model reaches higher spike rates of ∼79 Hz. At double the channel density of the original, the firing rate increases as the input rate increases smoothly with increasing channel density up to ∼69 Hz (Figure 5A). At low channel densities, a quarter of the original model, a peak in firing rate of ∼40 Hz occurs at a relatively low rate of synaptic inputs with a relatively small unitary conductance (Figure 5A). The maximum information rates occur at ∼100 inputs/second, irrespective of the channel density (Figure 5B). The information rate drops again as the synaptic input rate increases. At or below the original channel density, increasing the amplitude of synaptic inputs initially increases the information rate but then reduces it at higher synaptic amplitudes. At higher channel densities, increasing the synaptic amplitude does not produce a significant drop in information rate, reaching peak information rates of 292 bits/second (Figure 5B).

The energy consumption does not directly correspond to the firing rate (Figure 5C) because energy consumption incorporates the cost of producing a spike and the cost of postsynaptic potentials. Energy consumption increases with both rate and amplitude, irrespective of the channel density. However, the maximum energy consumption at the double channel density is 6.4 × 10^8^ ATP molecules/second, ∼3-fold higher than with a quarter the channel density of the original (Figure 5C). The energy efficiency was greatest at the lowest channel densities, with low inputs rates and small input amplitudes (Figure 5D). Increasing the rate or amplitude of the synaptic inputs reduced efficiency because it increases the energy consumption while simultaneously reducing the information rates.

## Discussion

We focused on how two interrelated factors, size and channel density, affect information coding in neuronal compartments, the energy cost it incurs, and the trade-off between performance and cost. Our simulations allowed us to explore the lower limits of biologically feasible compartments. Large compartments outperform small compartments at a given channel density, encoding more information per spike at a given spike rate. For a given compartment size, the original density of voltage-gated ion channels found in the squid giant axon maximizes the information rate. Although this may be a consequence of the coarseness with which we sampled channel densities, the same conclusion was reached by Schneidman *et al.*^25^

In larger compartments or at higher channel densities, the Na^+^/K^+^ pump must work harder to pump more ions across the membrane consuming more energy. Consequently, as the compartment size or channel density increases the cost per spike increases and the energy efficiency of information processing decreases. Double the original channel density consumes ∼74% more energy, while half consumes ∼42% less. Thus, although doubling or halving ion channel densities has a relatively small effect (double, 220 bits/second (2.3%); half, 218 bits/second (3.4%)) on the information rate,^25^ it has a significant effect on energy consumption. The substantial drop in energy consumption makes energy efficiency maximum at half the original channel density of the squid model despite the maximum information rate occurring at the original density. For a 50 *μ*m^2^ compartment, the maximum energy efficiency is attained at information rates ∼8.7% below the maximum achieved by the compartment.

The difference between the channel density that maximizes energy efficiency and that which maximizes information rate for a particular compartment size produces a conflict; information rate and energy efficiency cannot be maximized simultaneously. This conflict has been observed in models of spiking neurons and neural codes,^26, 27, 28^ adding to the numerous lines of evidence suggesting that energy is a selective pressure that has influenced the evolution of neural circuits.^29^ Thus, either size or channel density or both may be reduced to lower neuronal energy consumption. Yet they may not be reduced sufficiently to minimize energy consumption, or even energy efficiency; this will depend upon the amount of information required for generating adaptive behavior and the space available.^30^

Changing the rate and amplitude of the unitary synaptic conductances used to drive a model neuron also affects the information rate, energy consumption, and energy efficiency of the compartment. Maximum energy efficiency is achieved at consistently lower input rates and amplitudes than maximum information rates, again emphasizing that, in our models, they cannot be maximized simultaneously. Increasing channel density increases the spectrum across the input rates and amplitudes at which the maximum information rate is achieved. However, the compartments with high ion channel densities consume more energy and are less energy efficient. Thus, our simulations suggest that a trade-off exists among compartment size, channel density, and the rate and amplitude of the input stimulus. There is an input rate and amplitude that maximizes information rate for each combination of compartment size and channel density, but this differs from the rate and amplitude that maximizes energy efficiency.

Small compartments with low channel densities coding information at low spike rates from low-amplitude, sparse inputs are the most energy efficient. This suggests that to improve energy efficiency, information should be encoded by numerous small, low-firing-rate communication channels rather than a few large ones.^31^ This agrees with recent studies of the optic nerve that have shown that it saves energy by transmitting most of its information over low rate communication lines, using fine axons with small terminal arbors, and a smaller amount of information being transmitted in larger, high-rate axons.^13, 32^ A broader survey has suggested that this may be a common feature of numerous tracts in vertebrate and invertebrate nervous systems.^4^ However, our models address spike generation; the energy efficiency of the propagation of information along the axons requires deterministic and stochastic cable models.^6, 33^

### Why Does Membrane Area Constrain the Numbers of Channels Used for Signaling?

The voltage-gated ion channels, pumps and postsynaptic machinery of neurons must fit into a limited membrane area.^6^ Each channel/receptor or pump has a ‘footprint' that determines the minimum area occupied by a single molecule or synapse. Visual inspection of the structure of the voltage-gated K^+^ channel suggests it is a 40 × 40 Å structure.^34^ Assuming voltage-gated Na^+^ channels are similar in size, each single channel ‘footprint' is ∼1.6 × 10^−5^ *μ*m^2^. Thus, for a 300 *μ*m^2^ compartment based on the Hodgkin–Huxley squid giant axon model, ion channels consume an area of 0.37 *μ*m^2^, just over 0.1% of the surface area, leaving ∼299 *μ*m^2^ available for pumps and synapses.

Pumps have to occupy a major fraction of the remaining membrane area because they are slow. This is a major constraint. When a 300 *μ*m^2^ compartment is continuously stimulated with a synaptic input rate of 500 Hz in which each synaptic input increases the conductance by 0.2 mS/cm^2^, the Na^+^/K^+^ pump uses 1.07 × 10^9^ ATP molecules/second (Supplementary Table S3) to restore the ions that are passing through the voltage-gated, Leak, and synaptic conductances and generates a pump current of 172 pA. The maximal current a single Na^+^/K^+^ pump can generate is the product of its maximal turnover rate, ∼200 Hz, depending on temperature, voltage, and concentration,^35^ and the excess charge of one ion transported per cycle, ∼32 aA. Thus, a 300 *μ*m^2^ compartment needs approximately 5.4 million Na^+^/K^+^ pumps to generate a pump current of 172 pA. Because the Na^+^/K^+^ pump footprint is ∼4.9 × 10^−5^ *μ*m^2^ (∼70 × 70 Å),^36^ these Na^+^/K^+^ pumps occupy ∼262.15 *μ*m^2^ of membrane ∼88% of the surface area (c.f. 0.1% for voltage-gated channels). This leaves an area of 35.6 *μ*m^2^ in which to accommodate postsynaptic densities (PSDs). Assuming a diameter of 400 nm,^37^ a single PSD occupies an area of 0.13 *μ*m^2^. At a central glutamatergic synapse, a single PSD can contain ∼50 AMPA or NMDA receptors, sustaining around 2.5 nS of conductance change.^37^ The remaining 37.5 *μ*m^2^ can house 289 PSDs. Assuming that the 500 Hz synaptic input is generated by 50 PSDs, with each of their maximal signaling rate fixed at 10 Hz and conductance fixed at 2.5 nS, requires a total area of 6.5 *μ*m^2^ to accommodate the PSDs needed to generate a total conductance change of 0.6 pS.

Thus, to sustain the pump current in a 300 *μ*m^2^ compartment Na^+^/K^+^ pumps must consume >80% of the cell membrane. Although sufficient numbers of Na^+^/K^+^ pumps can be accommodated to meet demands at the original channel densities, our calculations show that there is insufficient space to meet demands with double the channel density (Supplementary Table S4). Consequently, at double the channel density, high spike rates could not be maintained indefinitely. This demonstrates that sustaining the Na^+^/K^+^ pump current constrains the combination of spike rate and channel density.

### Power Generation Limits the Numbers of Channels

In neurons, compartment sizes and channel numbers are also constrained by mitochondrial ATP synthesis. A neuron can accommodate only a limited number of mitochondria, restricting the rate of ATP production. Bottom-up estimates of the rate of mitochondrial ATP synthesis and, therefore, the volume of mitochondria needed to meet the energy demands of signaling, are hampered by the paucity of data available. However, Attwell and Laughlin^14^ estimate that the specific metabolic rate for a cortical neuron is ∼40 *μ*mol ATP/g per minute (based on measurements of the specific metabolic rate^38^ and assuming that one molecule of glucose yields 31 rather than 36 ATP molecules (due to the H^+^ Leak)). This gives a rate of ATP production of ∼4 × 10^5^ ATP molecules/*μ*m^3^ per second. Even assuming that the entire cell volume is composed of mitochondria, which is clearly an overestimate, none of the compartments we have modeled have enough volume to accommodate the mitochondria needed to power their Na^+^/K^+^ pumps (data not shown). Indeed, the power generated is approximately an order of magnitude less than would be required at the original channel density. This suggests that these compartments can only sustain firing rates substantially lower than those that our small model compartments generate. Thus, accommodating a sufficient volume of mitochondria is likely to limit the number of ion channels because of the power needed for the Na^+^/K^+^ pumps. Even when sufficient membrane area is available to accommodate the channels/receptors or pumps, there may be insufficient volume available for the mitochondria.

### Suitability of The Squid Giant Axon Model

We based our models on the squid giant axon model because the channel biophysics has been studied in detail^16, 17^ and because this model contains a single set of inward and outward voltage-dependent currents.^17^ Models containing channels with different kinetics and sensitivities will differ from our simulations in both information rates and energy consumption. In particular, the squid action potential consumes 67% to 94% more energy than many vertebrate action potentials because the voltage-gated Na^+^ and K^+^ currents overlap more than in any other known action potential. Consequently, the energy costs of many action potentials are considerably lower than our models predict.^23^ The high energy cost of the squid action potential will inflate our calculations of energy consumption, and the surface area and volume needed to support ion channels. Thus, considerably less surface area and volume may be required for low-cost action potentials such as those from cerebellar granule cells.^23^ In addition, the pronounced overlap of the inward and outward currents in the squid action potential may account for the strong dependency of energy efficiency on channel density. In general, the effects of changing the densities of both Na^+^ and K^+^ voltage-gated channels are model dependent. In some types of action potentials, efficiency decreases with density and in others it increases.^23^ Nonetheless, reducing compartment size reduces both costs and maximum information rates favouring the use of smaller neurons firing at lower rates.

### Limits to Compartment Size and Channel Density

We confirm that the spontaneous rate of action potentials increases dramatically as the compartments shrink, because smaller compartments have higher input resistances and this increases the probability that the spontaneous opening of Na^+^ channel triggers an action potential.^6, 24^ Small compartments, below 5 *μ*m in diameter, have spontaneous firing rates exceeding 10 Hz, increasing to 30 Hz below 2 *μ*m, irrespective of channel density. Because these compartments are so small, the cost of each action potential is low, however, our calculations suggest that there is an insufficient volume available to accommodate the mitochondria needed to power these action potentials. Moreover, spontaneous action potentials reduce the information rate by increasing noise entropy at the expense of signal entropy. Indeed, computational models of action potential propagation in narrow axons demonstrate that spontaneous activity generated by channel noise limits axon diameter.^6, 39^ Thus, despite the relatively high energy efficiencies of their action potentials, the limited power available and low information rates of small compartments may both prevent them from generating adaptive behavior. Very small compartments do exist in neurons^40^ (for a review, see Niven and Farris^30^). Little is known about the biophysical properties of the channels in these compartments but, if they use action potentials, they are likely to differ from those generated by larger neurons, including the squid giant axon.

## Conclusions

For a cell (compartment) containing ion channels from the squid giant axon of a given size, there is a channel density and an input stimulus that maximizes the energy efficiency of information coding. Changes in channel density and/or stimulus statistics can increase the information rate of the cell but only by sacrificing energy efficiency. This produces a Law of Diminishing Returns that penalizes excess capacity and promotes the reduction of channel density and input statistics to the minimum imposed by behavior. The available space within cells may also constrain spike rates and, consequently, information rates because of the space occupied by the number of Na^+^/K^+^ pumps needed to sustain firing and the mitochondria that power them. These findings emphasize the interactions between neuronal volume, membrane area, input statistics, and channel density, all of which affect the trade-off between energy consumption and information coding.
