# Supplementary figures and images for: The effect of cell size and channel density on neuronal information encoding and energy efficiency
Source: J Cereb Blood Flow Metab. 2013 Jun 19;33(9):1465–73. doi: 10.1038/jcbfm.2013.103 (PMC3764378; doi:10.1038/jcbfm.2013.103)

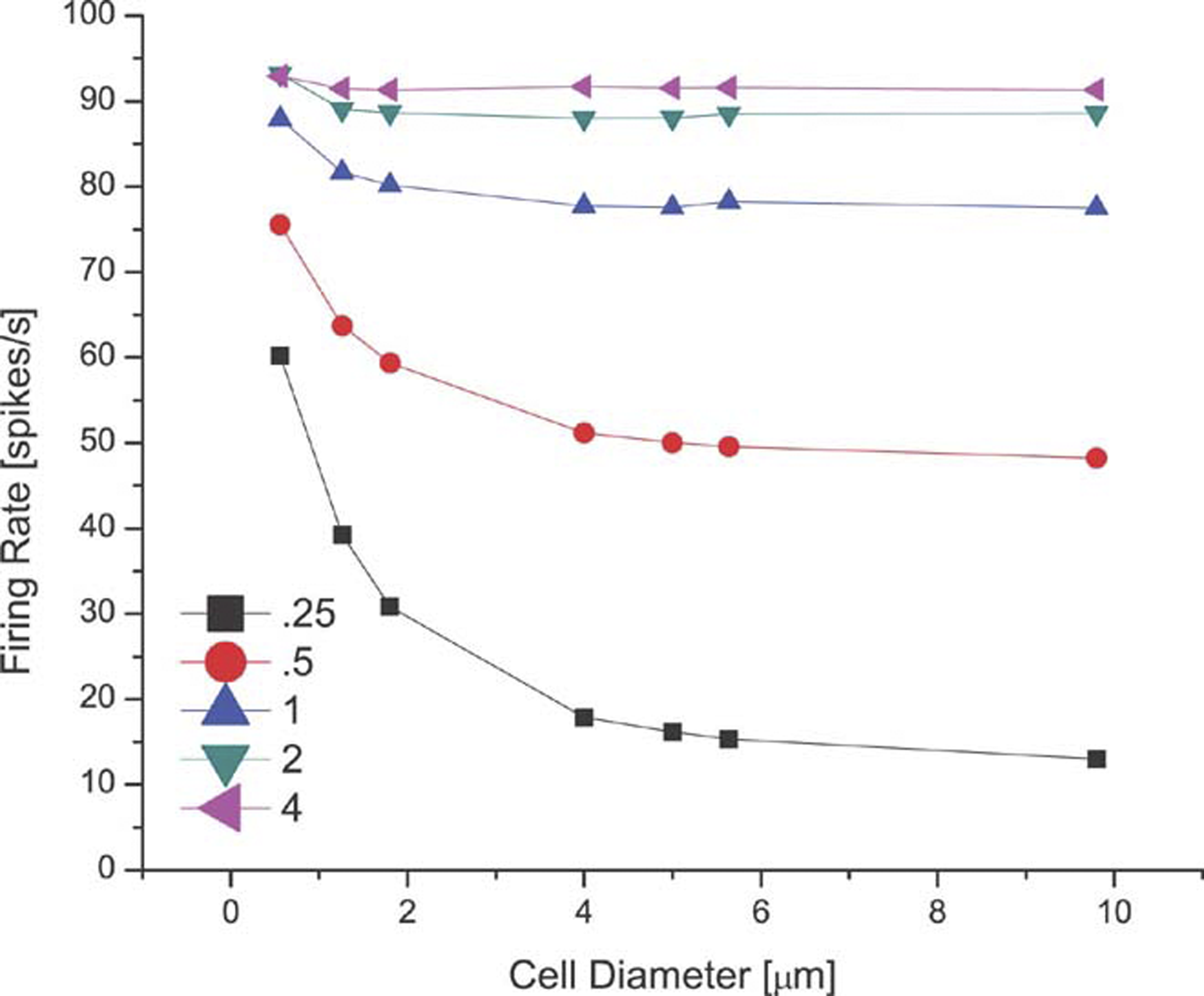

Supplement: Supplementary Figure 1 [file jcbfm2013103x1.tif]

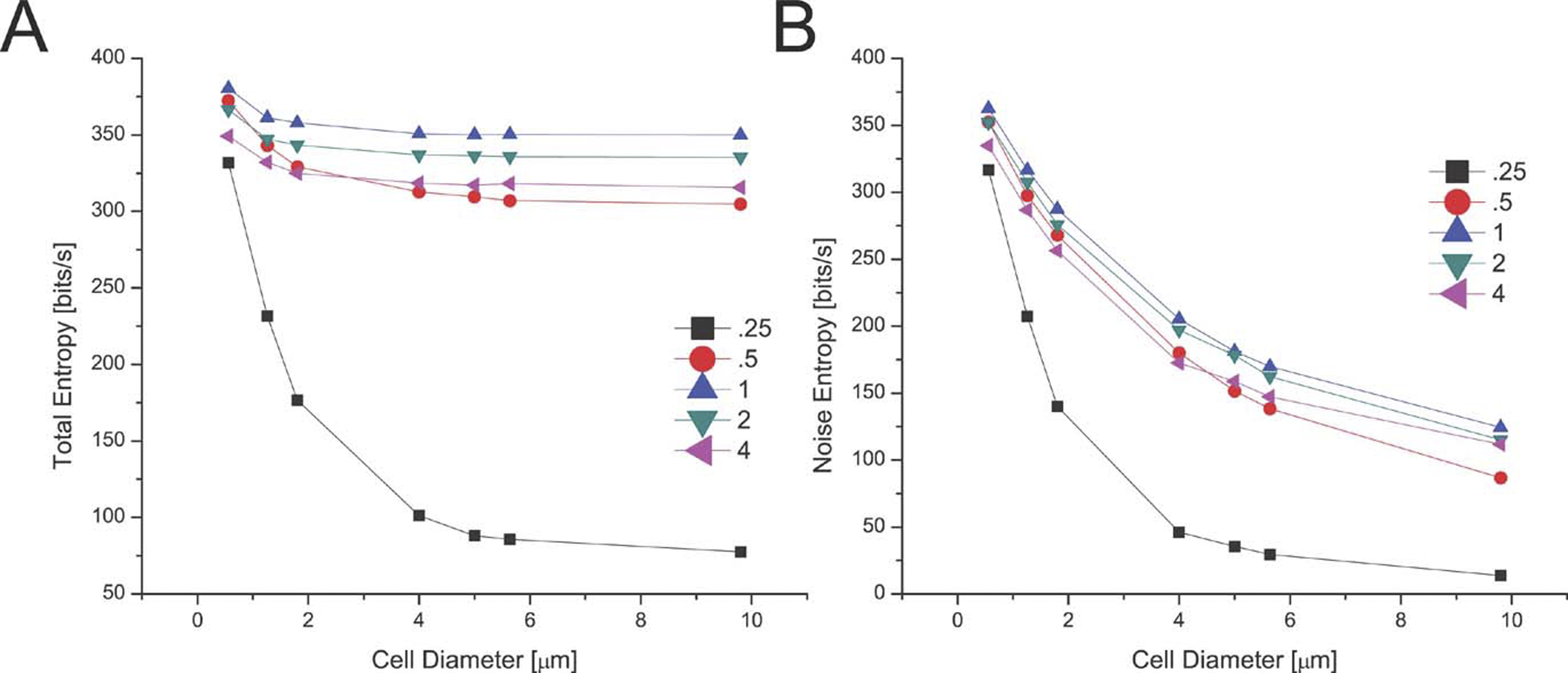

Supplement: Supplementary Figure 2 [file jcbfm2013103x2.tif]

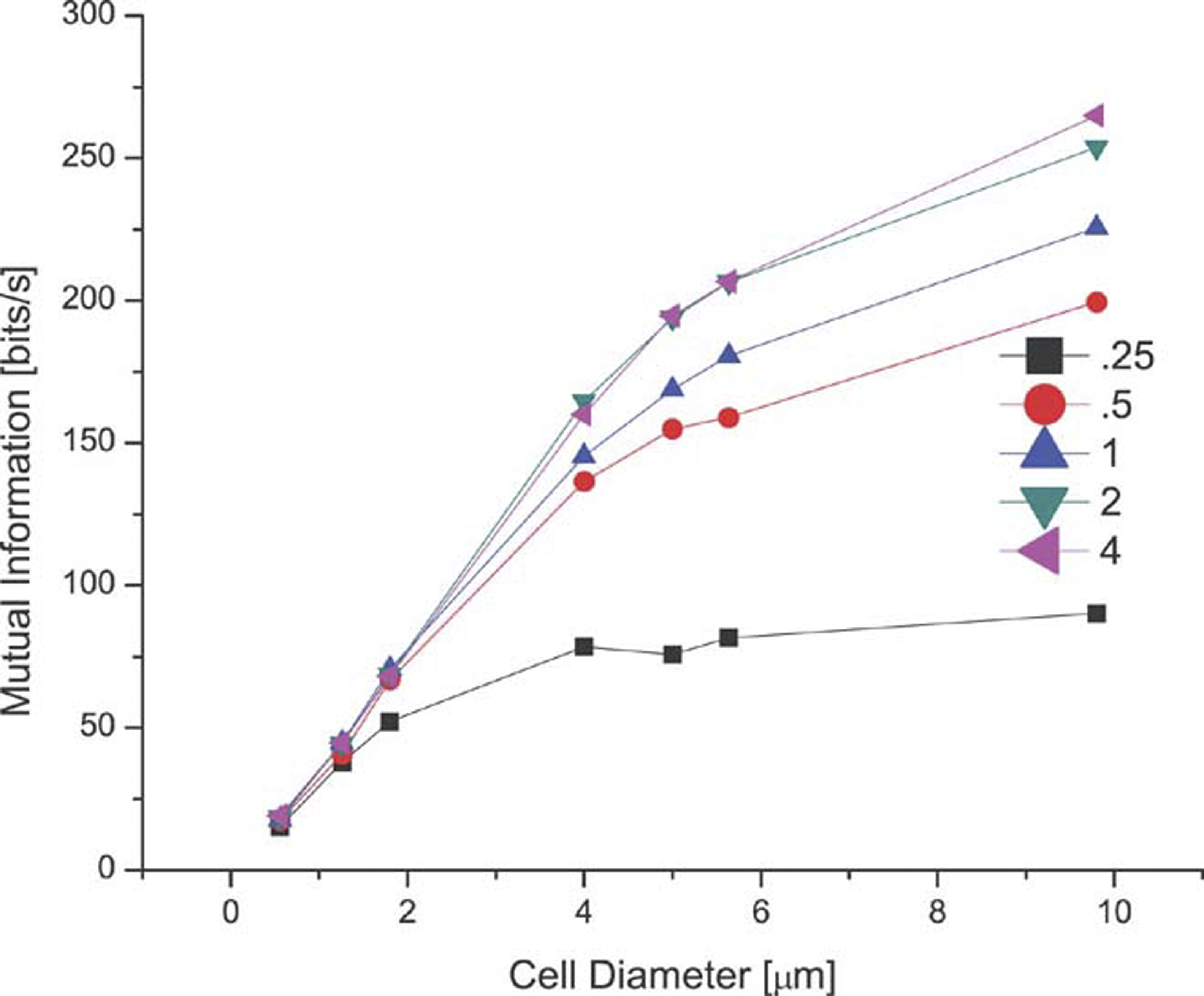

Supplement: Supplementary Figure 3 [file jcbfm2013103x3.tif]

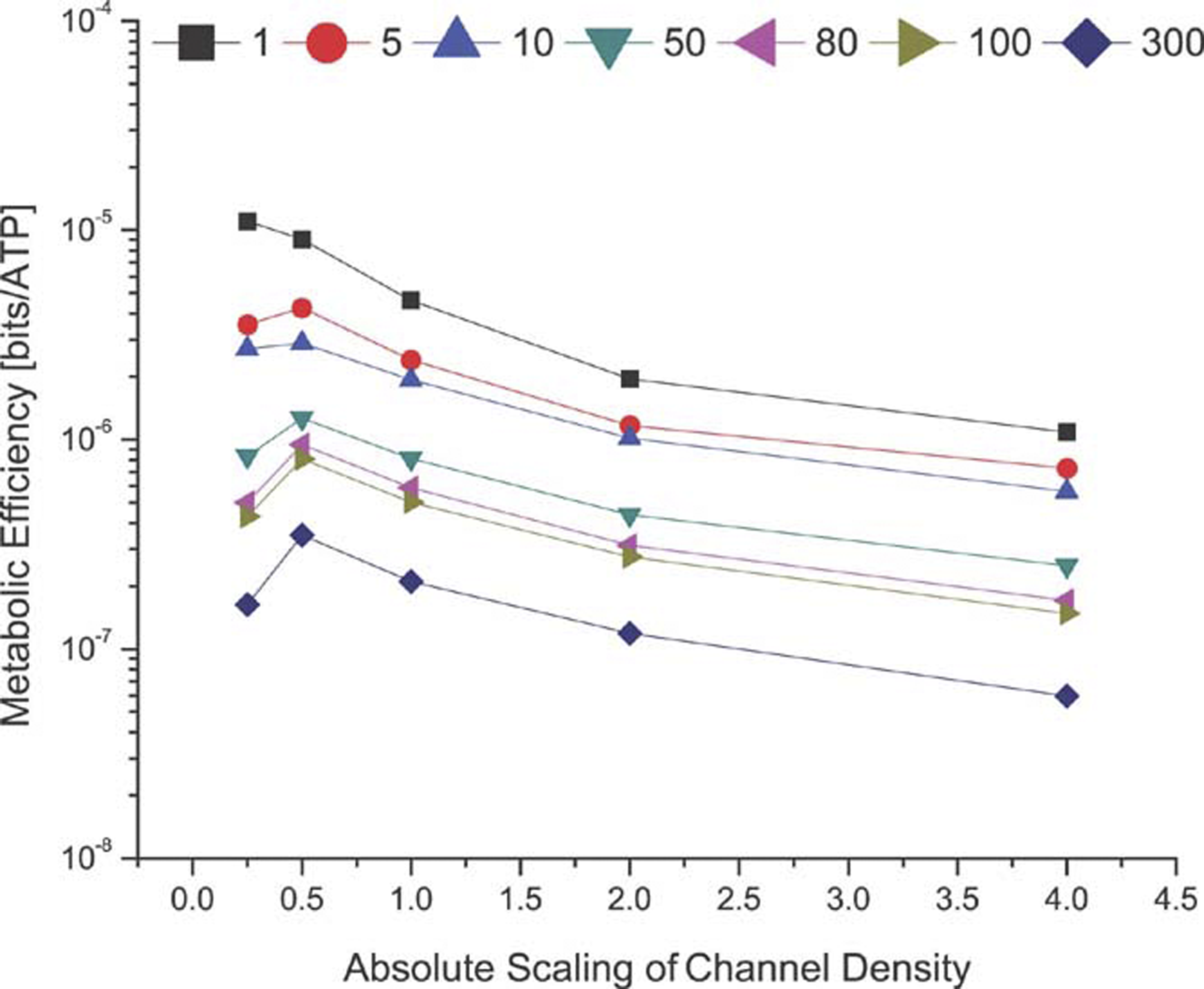

Supplement: Supplementary Figure 4 [file jcbfm2013103x4.tif]
