# Supplementary material for: The effect of cell size and channel density on neuronal information encoding and energy efficiency
Source: J Cereb Blood Flow Metab. 2013 Jun 19;33(9):1465–73. doi: 10.1038/jcbfm.2013.103 (PMC3764378; doi:10.1038/jcbfm.2013.103)
Supplement: Supplementary Tables [file jcbfm2013103x5.doc]

Table S1 Parameters for the stochastic Hodgkin-Huxley model

| SYMBOL | DEFINITION | VALUE, UNITS |
| --- | --- | --- |
| C_m_ | Specific membrane capacitance | $1\text{ }\mu F/cm^{2}$ |
| T | Temperature | $6.3\text{ }^{\circ}C$ |
| E_L_ | Leakage reversal potential | $-54.4mV$ |
| E_Na_ | Sodium reversal potential | $50\text{ }mV$ |
| E_K_ | Potassium reversal potential | $-77mV$ |
| g_Leak_ | Leakage conductance | $0.3\text{ }mS/cm^{2}$ |
| N_Na_ | Na^+^ channel density | $60/\mu m^{2}$ |
| N_K_ | K^+^ channel density | $18/\mu m^{2}$ |
| $\alpha_{m}(V)$ | Opening rate (activation, Na^+^) | $\frac{0.1(V+40)}{1-\exp\left(-0.1(V+40)\right)}$ |
| $\alpha_{h}(V)$ | Opening rate (inactivation, Na^+^) | $0.07\exp\left(-0.05\left(V+65\right)\right)$ |
| $\alpha_{n}(V)$ | Opening rate (activation, K^+^) | $\frac{0.01(V+55)}{1-\exp\left(-0.1(V+55)\right)}$ |
| $\beta_{m}(V)$ | Closing rate (activation, Na^+^) | $4\exp\left(-0.0556\left(V+65\right)\right)$ |
| $\beta_{h}(V)$ | Closing rate (inactivation, Na^+^) | $\frac{1}{1+\exp\left(-0.1(V+35)\right)}$ |
| $\beta_{n}(V)$ | Closing rate (activation, K^+^) | $0.125\exp\left(-0.0125\left(V+65\right)\right)$ |

Table S2 Underestimation of metabolic consumption (in %) when the flux induced by the leak current is ignored under absolute scaling of channel densities[[1]](#footnote-2).

| Cell Size  [μm^2^] | Quarter  [%] | Half  [%] | Original  [%] | Double  [%] | Quadruple  [%] |
| --- | --- | --- | --- | --- | --- |
| 1 | 42 | 16 | 6 | 3 | 1 |
| 5 | 32 | 14 | 6 | 3 | 1 |
| 10 | 35 | 15 | 7 | 3 | 1 |
| 50 | 59 | 24 | 11 | 5 | 2 |
| 80 | 80 | 33 | 15 | 7 | 3 |
| 100 | 91 | 40 | 18 | 9 | 4 |
| 300 | 122 | 79 | 49 | 30 | 19 |

Table S3 Trade-offs between numbers of voltage-gated ion channels, Na^+^/K^+^ pumps and synaptic sources

| Cell Size  [μm^2^] | Total channels | Channel  area  [μm^2^] | Remaining area  [μm^2^] | Empirical  consumption  [ATP/sec] | Pump  Current  [Ampere] | Pumps required | Pump area  [μm^2^] | Remaining area  [μm^2^] | Synaptic conductances (empirical)  [mS] | Possible PSDs | Synaptic conductances (plausible)  [mS] | Plausible/Actual |
| --- | --- | --- | --- | --- | --- | --- | --- | --- | --- | --- | --- | --- |
| 1 | 78 | 0.0012 | 0.999 | 3.86E+06 | 6.176E-13 | 1.9E+04 | 0.95 | 0.053 | 2.0E-09 | 0.42 | 1.06E-06 | 528 |
| 5 | 390 | 0.0062 | 4.994 | 1.86E+07 | 2.976E-12 | 9.3E+04 | 4.55 | 0.437 | 1.0E-08 | 3.48 | 8.69E-06 | 869 |
| 10 | 780 | 0.0125 | 9.988 | 3.67E+07 | 5.872E-12 | 1.8E+05 | 8.99 | 0.996 | 2.0E-08 | 7.93 | 1.98E-05 | 991 |
| 50 | 3900 | 0.0624 | 49.938 | 1.79E+08 | 2.864E-11 | 9.0E+05 | 43.85 | 6.083 | 1.0E-07 | 48.40 | 1.21E-04 | 1210 |
| 80 | 6240 | 0.0998 | 79.900 | 2.86E+08 | 4.576E-11 | 1.4E+06 | 70.07 | 9.830 | 1.6E-07 | 78.23 | 1.96E-04 | 1222 |
| 100 | 7800 | 0.1248 | 99.875 | 3.60E+08 | 5.76E-11 | 1.8E+06 | 88.20 | 11.675 | 2.0E-07 | 92.91 | 2.32E-04 | 1161 |
| 300 | 23400 | 0.3744 | 299.626 | 1.07E+09 | 1.712E-10 | 5.4E+06 | 262.15 | 37.476 | 6.0E-07 | 298.22 | 7.46E-04 | 1243 |

Table S4 Trade-offs between numbers of Na^+^/K^+^ pumps, synaptic sources with double the amount of voltage-gated Na^+^/K^+^ channels

| Cell Size  [μm^2^] | Total channels | Channel  area  [μm^2^] | Remaining area  [μm^2^] | Empirical  consumption  [ATP/sec] | Pump  Current  [Ampere] | Pumps required | Pump area  [μm^2^] | Remaining area  [μm^2^] | Synaptic conductances (empirical)  [mS] | Possible PSDs | Synaptic conductances (plausible)  [mS] | Is this setup plausible? |
| --- | --- | --- | --- | --- | --- | --- | --- | --- | --- | --- | --- | --- |
| 1 | 156 | 0.002496 | 0.997504 | 7.24E+06 | 1.16E-12 | 3.6E+04 | 1.773 | -0.775 | 2.0E-09 | -6.2 | -9.87E-05 | NO |
| 5 | 780 | 0.01248 | 4.98752 | 3.40E+07 | 5.44E-12 | 1.7E+05 | 8.332 | -3.345 | 1.0E-08 | -26.6 | -4.26E-04 | NO |
| 10 | 1560 | 0.02496 | 9.97504 | 6.64E+07 | 1.06E-11 | 3.3E+05 | 16.258 | -6.283 | 2.0E-08 | -50.0 | -8.00E-04 | NO |
| 50 | 7800 | 0.1248 | 49.8752 | 3.19E+08 | 5.11E-11 | 1.6E+06 | 78.180 | -28.304 | 1.0E-07 | -225.2 | -3.60E-03 | NO |
| 80 | 12400 | 0.1984 | 79.8016 | 5.07E+08 | 8.11E-11 | 2.5E+06 | 124.117 | -44.315 | 1.6E-07 | -352.6 | -5.64E-03 | NO |
| 100 | 15600 | 0.2496 | 99.7504 | 6.32E+08 | 1.01E-10 | 3.2E+06 | 154.816 | -55.065 | 2.0E-07 | -438.2 | -7.01E-03 | NO |
| 300 | 46800 | 0.7488 | 299.2512 | 1.86E+09 | 2.98E-10 | 9.3E+06 | 456.190 | -156.939 | 6.0E-07 | -1248.8 | -2.00E-02 | NO |

1. The percentage of overestimation is calculated as 100*(cost_active+leak_ – cost_active)/_ cost_active_. Simulations were done without any synaptic inputs. [↑](#footnote-ref-2)
