# Supplementary material for: The effect of cell size and channel density on neuronal information encoding and energy efficiency
Source: J Cereb Blood Flow Metab. 2013 Jun 19;33(9):1465–73. doi: 10.1038/jcbfm.2013.103 (PMC3764378; doi:10.1038/jcbfm.2013.103)
Supplement: Supplementary Figures Legends [file jcbfm2013103x6.doc]

**SUPPLEMENTARY FIGURE LEGENDS**

**Figure S1.**  The effect of changing the relative proportions of voltage-gated Na^+^ and K^+^ channels upon firing rate. The spike rates in model cells receiving excitatory synaptic inputs for ratio-metric scaling of voltage-gated Na^+^ and K^+^ channel densities from a quarter of to four times the density of those found in the squid giant axon. The values in the key on each graph correspond to the change in density of Na^+^ channels (>1) or K^+^ channels (<1) from that of the Hodgkin-Huxley squid giant axon model. 2 indicates double the density of voltage-gated Na^+^ channels compared to the original model, whist 0.25 indicates a four-fold increase in the density of K^+^ channels compared to the original model.

**Figure S2.** Total and noise entropy are affected by cell diameter and channel density. (A) As cell diameter increases, the total entropy drops. Channel densities higher or lower than the density of the original squid model reduce the total entropy, which is maximum for the original squid axon channel density for all cell diameters. (B) Irrespective of channel density, larger compartments have lower noise entropy than smaller compartments. The values in the key on each graph correspond to the change in channel density from that of the Hodgkin-Huxley squid giant axon model. 0.25 indicates a quarter the density of the original model, whist 2 indicates double the density of the original model.

**Figure S3.** The effect of changing the relative proportions of voltage-gated Na^+^ and K^+^ channels upon information coding. The mutual information for the compartments shown in S1. The values in the key on each graph correspond to the change in density of Na^+^ channels (>1) or K^+^ channels (<1) from that of the Hodgkin-Huxley squid giant axon model. 2 indicates double the density of voltage-gated Na^+^ channels compared to the original model, whist 0.25 indicates a four-fold increase in the density of K^+^ channels compared to the original model.

**Figure S4.** A channel density exists that maximises the energy efficiency of each compartment. For each compartment, except the smallest, the maximum occurs at half the channel density of the original squid giant axon model. The values in the key correspond to the area of the compartment being simulated from 1 to 300 µm^2^.
